# Functional Validation of Two Fungal Subfamilies in Carbohydrate Esterase Family 1 by Biochemical Characterization of Esterases From Uncharacterized Branches

**DOI:** 10.3389/fbioe.2020.00694

**Published:** 2020-06-26

**Authors:** Xinxin Li, Kelli Griffin, Sandra Langeveld, Matthias Frommhagen, Emilie N. Underlin, Mirjam A. Kabel, Ronald P. de Vries, Adiphol Dilokpimol

**Affiliations:** ^1^Fungal Physiology, Westerdijk Fungal Biodiversity Institute, Fungal Molecular Physiology, Utrecht University, Utrecht, Netherlands; ^2^Laboratory of Food Chemistry, Wageningen University & Research, Wageningen, Netherlands; ^3^Department of Chemistry, Technical University of Denmark, Lyngby, Denmark

**Keywords:** carbohydrate esterase, CAZy subfamilies, feruloyl esterase, acetyl xylan esterase, fungi, plant biomass degradation, ferulic acid

## Abstract

The fungal members of Carbohydrate Esterase family 1 (CE1) from the CAZy database include both acetyl xylan esterases (AXEs) and feruloyl esterases (FAEs). AXEs and FAEs are essential auxiliary enzymes to unlock the full potential of feedstock. They are being used in many biotechnology applications including food and feed, pulp and paper, and biomass valorization. AXEs catalyze the hydrolysis of acetyl group from xylan, while FAEs release ferulic and other hydroxycinnamic acids from xylan and pectin. Previously, we reported a phylogenetic analysis for the fungal members of CE1, establishing five subfamilies (CE1_SF1–SF5). Currently, the characterized AXEs are in the subfamily CE1_SF1, whereas CE1_SF2 contains mainly characterized FAEs. These two subfamilies are more related to each other than to the other subfamilies and are predicted to have evolved from a common ancestor, but target substrates with a different molecular structure. In this study, four ascomycete enzymes from CE1_SF1 and SF2 were heterologously produced in *Pichia pastoris* and characterized with respect to their biochemical properties and substrate preference toward different model and plant biomass substrates. The selected enzymes from CE1_SF1 only exhibited AXE activity, whereas the one from CE1_SF2 possessed dual FAE/AXE activity. This dual activity enzyme also showed broad substrate specificity toward model substrates for FAE activity and efficiently released both acetic acid and ferulic acid (∼50%) from wheat arabinoxylan and wheat bran which was pre-treated with a commercial xylanase. These fungal AXEs and FAEs also showed promising biochemical properties, e.g., high stability over a wide pH range and retaining more than 80% of their residual activity at pH 6.0–9.0. These newly characterized fungal AXEs and FAEs from CE1 have high potential for biotechnological applications. In particular as an additional ingredient for enzyme cocktails to remove the ester-linked decorations which enables access for the backbone degrading enzymes. Among these novel enzymes, the dual FAE/AXE activity enzyme also supports the evolutionary relationship of CE1_SF1 and SF2.

## Introduction

Over 5000 million tons of agro-food industrial side streams, such as wheat straw, rice straw, corn stover, potato peelings, and sugarcane bagasse, are produced from the agro-industry annually (Chandra et al., 2012). This plant biomass contains mainly polysaccharides, i.e., cellulose, hemicellulose and pectin, and the aromatic polymer lignin, which form a network of the plant cell wall and, therefore, provide protection against pathogens and pests (Ithal et al., 2007; Liu et al., 2018). Xylan is a major component of hemicellulose from agro-food industrial side streams, accounting for 20–30% in secondary cell wall of dicots and up to 50% in commelinid monocots (Scheller and Ulvskov, 2010). Xylan composes of a β-1,4-xylosyl backbone with different substituents, e.g., L-arabinose, D-galactose, D-(methyl)glucuronic acid, ferulic acid, and acetic acid. The acetyl groups are linked to the *O*-2 and/or *O*-3 position of the xylosyl units in commelinid monocots (cereals and grasses) (Puls, 1997; Appeldoorn et al., 2010, 2013), whereas feruloyl and to a lesser degree *p*-coumaryl groups are esterified mainly at the *O*-5 position on arabinosyl residues of xylan of commelinid monocots (Smith and Hartley, 1983; Saulnier et al., 1995; Wende and Fry, 1997; Schendel et al., 2016). Feruloyl groups are also present in pectin, which esterified to the *O*-2 and/or *O*-5 positions of arabinan side chains and to the *O*-6 position of D-galactosyl residues in (arabino-)galactan of rhamnogalacturonan I (Carpita, 1996; Levigne et al., 2004; Harris, 2005; Harris and Trethewey, 2010). Acetyl xylan esterases (AXEs, EC 3.1.1.72) catalyze the hydrolysis of ester linkages between acetyl groups and xylan, whereas feruloyl esterases (FAEs, EC 3.1.1.73) hydrolyze the ester linkages between hydroxycinnamic acids and plant cell-wall polysaccharides (Christov and Prior, 1993).

The Carbohydrate Active enZyme (CAZy) database classifies carbohydrate esterases into 17 families (CE1–CE17) (Lombard et al., 2013). The characterized fungal AXEs belong to CE1-CE6 and CE16 families, with the majority of the characterized enzymes in CE1. Part of the characterized fungal FAEs are also assigned to CE1, whereas the other FAE subfamilies are not classified in this database (Dilokpimol et al., 2016). Recently, a phylogenetic classification of the fungal members of CE1 was established, which divided the fungal CE1 members into five subfamilies (CE1_SF1-SF5) (Mäkelä et al., 2018). The characterized AXEs were grouped in CE1_SF1 and the characterized FAEs were in CE1_SF2 and SF5, whereas none of the members from CE1_SF3 and SF4 has been characterized so far. Among these subfamilies, CE1_SF1 and SF2 are more related and originated from the same node, but target substrates with a different molecular structure. In this study, we aimed to explore the differences between CE1_SF1 and SF2 by selecting six ascomycete candidates from the uncharacterized brunches of the CE1_SF1 and SF2 for heterologous production in *Pichia pastoris* and biochemical characterization using different model and plant biomass substrates.

## Materials and Methods

### Materials

Methyl *p*-coumarate, methyl caffeate, methyl ferulate, methyl sinapate, ethyl *p*-coumarate, ethyl ferulate and chlorogenic acid were purchased from Apin Chemicals Limited (Oxon, United Kingdom). Insoluble wheat arabinoxylan (WAX, P-WAXYI, from wheat flour), endo-α-(1→5)-arabinanase (E-EARAB, GH43 from *Aspergillus niger*), and endo-β-(1→4)-galactanase (E-EGALN, GH53 from *A. niger*) were from Megazyme (Wicklow, Ireland). Corn oligosaccharides mix (COS) was provided by Wageningen University (Appeldoorn et al., 2013). Wheat bran (WB) was from Wageningen Mill (Wageningen, Netherlands). Corn xylooligosaccharides mix (CX) was from Carl Roth GmbH + Co. KG (Karlsruhe, Germany). Sugar beet pectin (SBP, Pectin Betapec RU 301) was from Herbstreith & Fox KG (Neuenbürg, Germany). Xylanase (GH11 from *Thermomyces lanuginosus*), *p*-methylumbelliferyl acetate (MUB-acetate) and other chemicals were purchased from Sigma-Aldrich (Merck KGaA, Darmstadt, Germany).

### Bioinformatics

All amino acid sequences in this study were obtained from JGI Mycocosm^1^ (Grigoriev et al., 2014) and the CAZy database^2^ (Lombard et al., 2013). The secretory signal peptides were detected using the SignalP 4.1^3^ (Petersen et al., 2011). The amino acid sequences without predicted signal peptides were aligned using Multiple Alignment using Fast Fourier Transform (MAFFT)^4^ (Katoh and Standley, 2013) and visualized using Easy Sequencing in Postscript^5^ (Robert and Gouet, 2014). The phylogenetic analysis was performed using maximum likelihood (ML), neighbor-joining (NJ), and minimal evolution (ME) implemented in the Molecular Evolutionary Genetic Analysis software version 7 (MEGA7)^6^ (Kumar et al., 2016) with 95% partial deletion and the Poisson correction distance of substitution rates. Statistical support for phylogenetic grouping was estimated by 500 bootstrap re-samplings. The final phylogenetic tree was shown using the ML tree with above 40% bootstrap next to the branches. *N*-glycosylation sites were predicted using NetNGlyc 1.0^7^ (Blom et al., 2004). The theoretical molecular masses were calculated from the amino acid sequences without signal peptide using Sequence Manipulation Suite online tool^8^ (Kearse et al., 2012).

### Cloning and Transformation to *Pichia pastoris*

The selected candidate genes (JGI accession numbers are provided in Table 1) without predicted signal peptide and introns were codon optimized and synthesized into pPicZαA plasmid for production in *P. pastoris* (Genscript Biotech, Leiden, Netherlands). The pPicZαA containing synthetic genes were transformed into *Escherichia coli* DH5α for propagation and sequencing. Then, the plasmids were extracted, linearized by *PmeI* (Promega, Madison, WI, United States), and transformed into *P. pastoris* strain X-33 (Invitrogen, Thermo Fisher Scientificı, Carlsbad, CA, United States) according to the manufacturer’s recommendation (Dilokpimol et al., 2017). The positive colonies were selected for the highest protein production based on colony Western Blot using anti Histidine-tag antibody conjugated with alkaline phosphatase (Thermo Fisher Scientific). The transformants were grown on a nitrocellulose membrane (0.45 μm; Whatman, GE Healthcare Life Sciences, Buckinghamshire, United Kingdom) over minimal medium (MM, 1.34% yeast nitrogen base, 4 × 10^–5^% biotin, 1% v/v methanol and 1.5% agar) for 2–4 days at 30°C. Then the membrane was washed with milli-Q water, blocked with 2–5% skim milk in phosphate-buffered saline, and blotted with anti Histidine-tag antibody. The signal was detected using 5-bromo-4-chloro-3-indolyl phosphate/nitro blue tetrazolium system.

**TABLE 1:** Molecular mass and properties of characterized CE1_SF1 and SF2 and the selected enzymes in this study.

SF	Fungal species	GenBank or JGI Accession number	Enzyme Name	Calculated molecular mass (kDa)	Apparent molecular mass (kDa)^a,b^	pH optimum^a^	pH stability^a^	Temp. optimum (°C)^a^	Temp. stability (°C)^a^	References
1.1	*Aspergillus luchuensis*^c^	BAA13434.2	*Al*AXEA (*Aw*AXE)	33	30	7.0	6.0–9.0	N.A.	40	(Koseki et al., 1997; Komiya et al., 2017)
1.1	*Aspergillus niger*	CAA01634.1	AxeA	33	N.A.	N.A.	N.A.	N.A.	N.A.	(De Graaff et al., 1992)
1.1	*Aspergillus ficuum*	AAK60128.1	*Af*AXE	33	34	7.0	N.A.	37	50	(Chung et al., 2002)
1.1	*Aspergillus oryzae*	jgi| Aspor1| 10625	*Ao*AXE	34	30	6.0	6.0–7.0	45	40–50	(Koseki et al., 2006)
1.1	*Aspergillus nidulans*	jgi| Aspnid1| 4188	*An*AXE	34	N.A.	N.A.	N.A.	N.A.	N.A.	(Bauer et al., 2006)
1.1	*Thermothelomyces thermophilus*^d^	ADZ98864.1	*Mt*Axe3	35	N.A.	7.0	N.A.	35–45	N.A.	(Pouvreau et al., 2011)
1.1	*Rasamsonia emersonii*	ADX07526.1	*Te*CE1	30	33	N.A.	N.A.	N.A.	N.A.	(Neumüller et al., 2015)
**1.2**	***Podospora anserina* S mat+**	**jgi| Podan2| 8904**	**AxeA**	**32**	**35**	**7.0**	**6.0–9.0^f^**	**40**	**40^f^**	**This study**
**1.3**	***Parastagonospora nodorum* SN15^e^**	**jgi| Stano2| 8578**	**Axe1**	**35**	**35**	**7.0**	**6.0–9.0^f^**	**30**	**40^f^**	**This study**
**1.3**	***Podospora anserina* S mat+**	**jgi| Podan2| 6933**	**AxeB**	**45**	**70**	**6.0**	**6.0–9.0^f^**	**40**	**30^f^**	**This study**
1.4	*Volvariella volvocea*	jgi| Volvo1| 112494	*Vv*AXE1	38	45	8.0	N.A.	60.0	40–50	(Ding et al., 2007)
1.4	*Phanerochaete chrysosporium*	AEX99751.1	*Pc*Axe2	40	55	7.0	N.A.	30–35	N.A.	(Huy et al., 2013)
**2.1**	***Podospora anserina* S mat+**	**jgi| Podan2| 8825**	**FaeD**	**33**	**35**	**7.0**	**3.0–10.0^f^**	**50**	**40^f^**	**This study**
2.2	*Penicillium funiculosum*	CAC14144.1	FAEB	39	35	N.A.	N.A.	N.A.	N.A.	(Kroon et al., 2000)
2.2	*Aspergillus sydowii*	jgi| Aspsy1| 1158585	*As*FaeE	33	32	N.A.	N.A.	N.A.	N.A.	(Dilokpimol et al., 2018)
2.2	*Neurospora crassa*	jgi| Neucr2| 3766	*Nc*Fae-1	32	29	6.0	6.0–7.5	55	N.A.	(Crepin et al., 2003)
2.2	*Chaetomium sp.*	AFU88756.1	*Chae*Fae	33	30	7.5	4.0–10.0	60	55	(Yang et al., 2013)
2.2	*Thermothelomyces thermophilus*^d^	AEO62008.1	*Mt*Fae1a	33	30	7.0	7.0–10.0	50	55	(Topakas et al., 2012)
2.2	*Chrysosporium lucknowense*	AEP33618.1	*Cl*FaeB2	33	33	7.0	N.A.	45	N.A.	(Kühnel et al., 2012)

*^*a*^ N.A., not available. ^*b*^ Apparent mass after deglycosylation by Endoglycosidase H. ^*c*^Formerly *Aspergillus awamori.*^*d*^Formerly *Myceliophthora thermophila.*^*e*^Formerly *Stagonospora*/*Septoria/nodorum.*^*f*^ Retaining more than 80% residual activity after incubating at 30°C or at pH 6.0 for pH or temperature stability, respectively. Enzymes in bold are used in this study.*

### Production of Recombinant Proteins

The selected *P. pastoris* transformants were grown according to Knoch et al. (2013) in a buffered complex glycerol medium (BMGY, 1% yeast extract, 2% peptone, 0.1 M potassium phosphate buffer pH 6.0, 1% w/v glycerol, 1.34% yeast nitrogen base, 4 × 10^–5^% biotin) overnight at 30°C, 250 rpm. Induction was done in buffered complex methanol medium (BMMY, BMGY without glycerol) at 22°C, 250 rpm with methanol supplemented (1% v/v) every 24 h for 96 h. Culture supernatants were harvested (4000 × *g*, 4°C, 20 min), filtered (0.22 mm; Merck), aliquoted and stored at −20°C prior further analysis. The stability of the recombinant enzymes was assessed by their activity (see below) each time after thawing. The enzyme activity remained more than 95% of their original activity while performing the experiment (data not shown).

### Biochemical Properties of Recombinant Proteins

Molecular masses of the recombinant proteins were estimated by SDS-PAGE (12% w/v, sodium dodecyl sulfate-polyacrylamide gel) using Mini-PROTEAN^®^ Tetra Cell (Bio-Rad, Hercules, CA, United States). Deglycosylation was performed by incubating 20 μL of *P. pastoris* culture supernatant with endoglycosidase H (New England Biolabs, Ipswich, MA, United States) as recommended by the manufacturer. Protein concentration was assessed from SDS-PAGE gel by densitometric method using ImageJ program (Schneider et al., 2012) and 0.5–2.0 μg Bovine Serum Albumin (Pierce, Thermo Scientific, Carlsbad, CA, United States) as a standard.

### Enzyme Activity Assays

#### *p*-Methylumbelliferyl Substrate Assay

Activity toward *p*-methylumbelliferyl acetate (MUB-acetate) was performed in 100 μL reaction mixtures containing 10 μM MUB- acetate (dissolved in acetone), 100 mM phosphate buffer (pH 6.0) and 10 μL culture supernatant. The reaction was performed at 30°C. The release of umbelliferone group was spectrophotometrically quantified by following the excitation/emission at 340/520 nm up to 30 min with a 2 min interval. The activity of the enzymes was determined by quantification of the umbelliferone group using a standard curve (0.5–100 μM). One unit (U) of AXE activity was defined as the amount of enzyme which released 1 μmol of umbelliferone group from MUB-acetate per min under the assay condition. Culture filtrate from *P. pastoris* harboring pPicZαA plasmid without insertion was used as negative control. All assays were performed in triplicate.

#### Hydroxycinnamate Substrate Assay

Activity of the CE1_SF1 and SF2 candidates toward hydroxycinnamate substrates (methyl *p*-coumarate, methyl caffeate, methyl ferulate, methyl sinapate, ethyl *p*-coumarate, ethyl ferulate and chlorogenic acid) was performed in 250 μL reaction mixtures containing 0.12 mM substrate (dissolved in dimethylformamide), 80 mM phosphate buffer (pH 6.0) and 50 μL culture supernatant. The decrease of substrate concentration was spectrophotometrically quantified by following the absorbance at 340 nm up to 30 min with a 2 min interval. The activity of the enzymes was determined by quantification of the substrate concentration using standard curves in a range between 0.005 and 0.25 mM. One unit (U) of FAE activity was defined as the amount of enzyme which decreased 1 μmol of substrates per min under the assay condition. Culture filtrate from *P. pastoris* harboring pPicZαA plasmid without insertion was used as negative control. All assays were performed in triplicate.

#### pH and Temperature Profiles

*p*-methylumbelliferyl-acetate and methyl ferulate were used as substrates for AXE and FAE activity, respectively, based on the above assay. pH profiles of enzyme activity were determined at 30°C in 100 mM Britton-Robinson buffer (Britton and Robinson, 1931) (pH 2.0–9.0). The temperature profiles of enzyme activity were measured in 100 mM phosphate buffer pH 6.0 at 22–80°C. The pH and thermal stability of the enzymes were determined by measuring the residual enzyme activities after 2 or 16 h incubation at 30°C in 100 mM Britton-Robinson buffer pH 2.0–10.0, or after 2 h incubation at 22 to 80°C in phosphate buffer pH 6.0 (Dilokpimol et al., 2011).

### Hydrolytic Activity Toward (poly- /oligo-)saccharides

The activity toward plant biomass was determined using insoluble wheat arabinoxylan (WAX) and corn oligosaccharides mix (COS) for AXE activity, and WAX, wheat bran (WB), corn xylooligosaccharides mix (CX), and sugar beet pectin (SBP) for FAE activity. For pre-treatment, 1% of WAX, COS, WB, and CX was incubated with 0.1 mg xylanase, or 0.1 mg of arabinanase and galactanase for SBP, in a 50 mM sodium acetate buffer pH 4.5 and 0.02% sodium azide at 100 rpm and 30°C for 72 h. The reaction was stopped by heat inactivation at 95°C for 10 min (Dilokpimol et al., 2017). For hydrolytic activity assay, the reaction containing 500 μL 1% (pre-treated) substrate and 100 μL culture supernatant (containing 1 μg enzyme) was incubated at 30°C, 24 h, 100 rpm. Except for COS reactions that were stopped by the addition of 50 μL 2 M HCl, the others were inactivated by heating at 95°C, 10 min and centrifuged at 14,000 rpm, 4°C for 15 min. The supernatants were used for HPLC analysis.

#### Acetic Acid Content Analysis

The acetic acid release was measured by using HPLC (Dionex ICS-3000 chromatography system; Thermo Scientific, Sunnyvale, CA, United States) equipped with an Aminex HPX-87H column with a guard-column (300 × 7.8 mm; Bio-Rad) and a refractive index detector (Bio-Rad). An isocratic elution of 5.0 mM sulfuric acid, with a flow rate of 0.6 mL/min at 40°C was used according to Zeppa et al. (2001). The release of acetic acid was quantified using standards in a range between 0.01 and 2.0 mg/mL.

#### Hydroxycinnamic Acid Content Analysis

The reaction supernatants were mixed with 100% acetonitrile (1:3, v/v), then incubated on ice for 10 min and centrifuged for 15 min at 4°C to remove precipitants prior to the analysis. The ferulic release was monitored by using HPLC (Dionex ICS-5000+ chromatography system; Thermo Scientific, Sunnyvale, CA) equipped with an Acclaim Mixed-Mode WAX-1 LC Column (3 × 150 mm; Thermo Scientific) and a UV detector (310 nm; Thermo Scientific). The chromatographic separation was carried out according to Dilokpimol et al. (2017) using an isocratic elution of 25 mM potassium phosphate buffer, 0.8 mM pyrophosphate, pH 6.0 in 50% (v/v) acetonitrile with a flow rate of 0.25 mL/min at 30°C. Ferulic and *p*-coumaric acids (0.25–50 μM) were used as standards for identification and quantification.

## Results

### Production of Four New Fungal CE1 Enzymes

**FIGURE 1:**
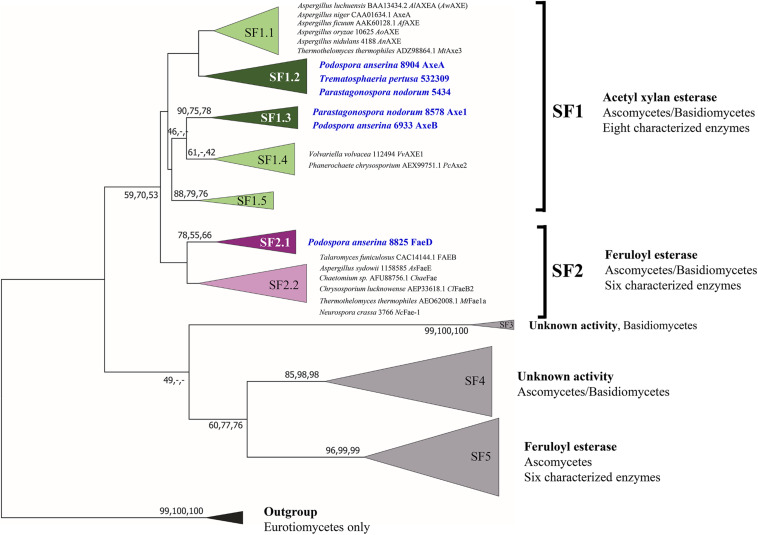
Reconstructed phylogenetic tree of fungal CE1 members [modified from Mäkelä et al. (2018)]. The phylogenetic analysis was performed by maximum likelihood (ML) implemented in MEGA7 (Kumar et al., 2016) with 95% partial deletion of gaps and the Poisson correction distance of substitution rates. The main branches/subfamilies were collapsed. Statistical support for phylogenetic grouping was estimated by 500 bootstrap re-samplings, only the bootstrap above 40% were shown on the branches, also of a neighbor-joining (NJ) and minimal evolution (ME) tree using the same dataset (order: ML, NJ, ME). Eight FAEs from subfamily 7 (Dilokpimol et al., 2016) were used as an outgroup. Names in blue and bold indicates the selected candidates for this study. SF, subfamily. The full phylogenetic tree can be found in Supplementary Figure 1.

Previous phylogenetic tree for fungal CE1 was proposed which classified the members of CE1 into five subfamilies (Mäkelä et al., 2018). Using a genome-mining strategy by BLAST searching against 18 genome sequenced fungal species (Dilokpimol et al., 2018), we added 87 additional CE1 sequences to the previous CE1 tree (Mäkelä et al., 2018), and reconstructed the phylogenetic tree (Figure 1, Supplementary Figure 1, and Supplementary Table 1). The reconstructed CE1 tree is agreed with the previous one. CE1_SF1 could be further separated into five branches (CE1_SF1.1–SF1.5), while CE1_SF2 was divided into two branches (CE1_SF2.1–SF2.2). Fungal CE1_SF1 contains nine biochemically characterized AXEs from CE1_SF1.1 and SF1.4, while fungal CE1_SF2 contains six biochemically characterized FAEs that were all from CE1_SF2.2 (Figure 1 and Table 1). It is possible that the uncharacterized branches contain enzymes with different activity. To systematically evaluate the subfamilies and compare the activities and biochemical properties, six fungal candidates from the uncharacterized branches of these two subfamilies (three from CE1_SF1.2, two from CE1_SF1.3 and one from CE1_SF2.1) were selected for recombinant protein production in *P. pastoris*.

Of these six candidates, only four candidate enzymes, i.e., *Podospora anserina* AxeA, AxeB, *Parastagonospora nodorum* Axe1, and *Po. anserina* FaeD, were detected by SDS-PAGE (Supplementary Figure 2). The apparent masses of *Pa. nodorum* Axe1, *Po. anserina* AxeB, and *Po. anserina* FaeD were 40, 100, and 45 kDa, respectively, whereas *Po. anserina* AxeA showed smears. After deglycosylation using endoglycosidase H, the molecular masses of *Po. anserina* AxeA, *Pa. nodorum*, Axe1, and *Po. anserina* FaeD decreased to 35, 35, and 35 kDa, respectively, which corresponded with their calculated masses based on the amino acid sequence. In contrast, the deglycosylation of *Po. anserina* AxeB resulted in a molecular mass of about 70 kDa, which was still higher than the calculated molecular mass. It is possible that the glycosylation sites of *Po. anserina* AxeB were inaccessible for endoglycosidase H or that other post-translational modifications were present.

### All Four New Enzymes Have High Alkaline Tolerance

*Podospora anserina* AxeA, AxeB, *Pa. nodorum* Axe1 were active toward MUB-substrates, while *Po. anserina* FaeD showed a broad substrate range. It hydrolyzed all tested substrates and showed the highest activity toward methyl *p*-coumarate (141.5 ± 3.5 U/mg) (Table 2).

**TABLE 2:** Specific activity of positive candidates toward model substrates.

Fungal species	Enzyme	Concentration	Specific activity (U/mg)^a^							
		
		(mg/L)	MUB-acetate	Methyl *p*-coumarate	Methyl caffeate	Methyl ferulate	Methyl sinnapate	Ethyl *p*-coumarate	Ethyl ferulate	Chlorogenic acid
*Podospora anserina*	AxeA	12	30.4 ± 0.4	N.A.	N.A.	N.A.	N.A.	N.A.	N.A.	N.A.
*Parastagonospora nodorum*	AxeB	21	33.4 ± 0.3	N.A.	N.A.	N.A.	N.A.	N.A.	N.A.	N.A.
*Podospora anserina*	Axe1	55	22.1 ± 0.0	N.A.	N.A.	N.A.	N.A.	N.A.	N.A.	N.A.
*Podospora anserina*	FaeD	73	3.1 ± 0.1	141.5 ± 3.5	53.0 ± 0.3	49.8 ± 0.3	50.0 ± 0.1	48.1 ± 2.1	39.2 ± 0.6	22.8 ± 1.4

*^*a*^N.A., not active.*

**FIGURE 2:**
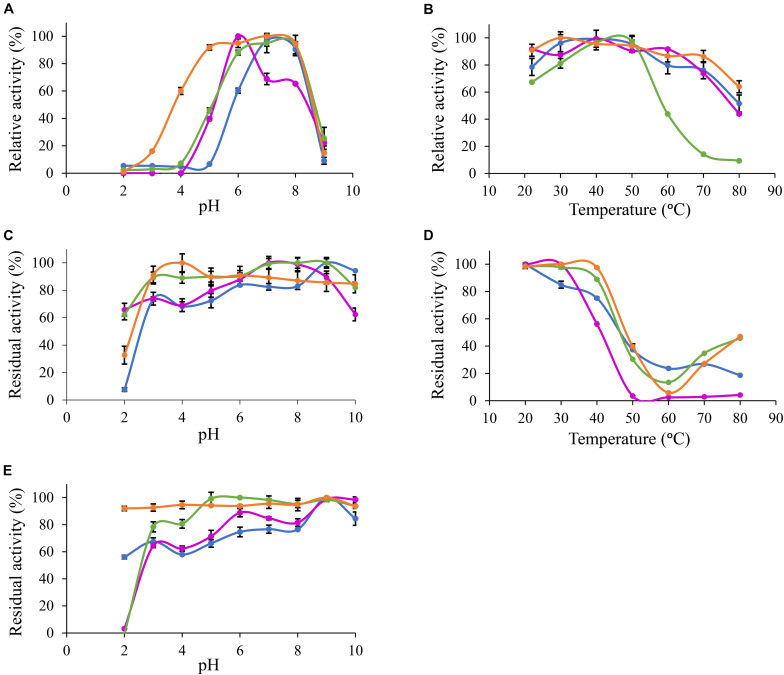
pH and temperature profiles of the selected CE1 enzymes toward *p*-methylumbelliferyl acetate. **(A)** pH optimum, **(B)** Temperature optimum, **(C)** pH stability for 2 h incubation at 30°C, **(D)** Temperature stability for 2 h incubation at pH 6.0, **(E)** pH stability for 16 h incubation at 30°C. 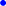 *Po. anserina* AxeA; 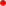 *Pa. nodorum* Axe1; 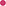 *Po. anserina* AxeB; 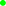 *Po. anserina* FaeD. Each experiment was performed in triplicate. Standard deviations are shown as error bars.

Based on MUB-acetate, *Po. anserina* AxeA, AxeB, *Pa. nodorum* Axe1 and *Po. anserina* FaeD showed the highest activity at pH 7.0, 6.0, 7.0, and 7.0, respectively, when incubated at 30°C (Figure 2A). They showed the highest activity at 40, 40, 50, and 30°C, respectively, when incubated at pH 6.0 (Figure 2B). The enzymes showed stability over a wide pH range, retaining more than 80% residual activity after incubation at pH 6.0–9.0 for 2 h (Figure 2C). In addition, *Pa. nodorum* Axe1 and *Po. anserina* FaeD retained more than 85% residual activity between pH 3.0 and 10.0. The stability of *Po. anserina* AxeA and *Pa. nodorum* Axe1 was higher, since both enzymes retained 55 and 90% of their residual activity at pH 2, compared to *Po. anserina* AxeB and *Po. anserina* FaeD, which did not show any residual activity after incubation for 16 h (Figure 2E). Most enzymes retained more than 80% residual activity after incubation at 40°C for 2 h except for *Po. anserina* AxeB that retained only 55% residual activity (Figure 2D).

**FIGURE 3:**
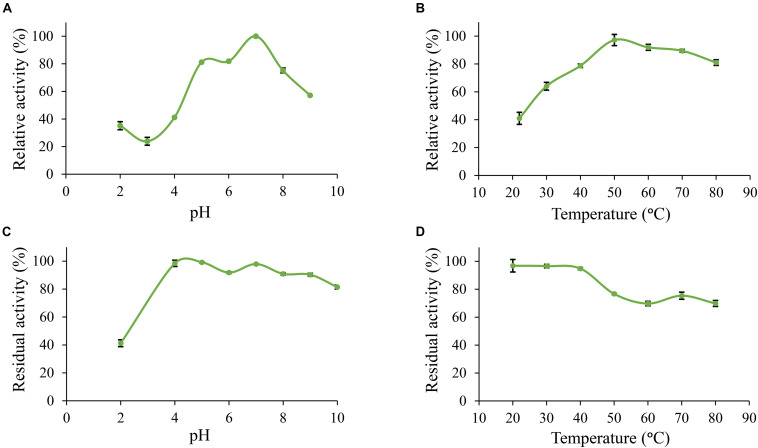
pH and temperature profiles of *Po. anserina* FaeD toward methyl ferulate. **(A)** pH optimum, **(B)** Temperature optimum, **(C)** pH stability for 2 h incubation at 30°C, **(D)** Temperature stability for 2 h incubation at pH 6.0. Each experiment was performed in triplicate. Standard deviations are shown as error bars.

Furthermore, *Po. anserina* FaeD showed the highest activity toward methyl ferulate at pH 7.0 and 50°C (Figures 3A,B). It retained more than 80% residual activity after incubation at pH 4.0–10.0 for 2 h (Figure 3C). *Po. anserina* FaeD also retained more than 70% residual activity after incubation at 80°C for 2 h (Figure 3D).

### *Po. anserina* FaeD Is a Dual FAE/AXE Activity Enzyme

**FIGURE 4:**
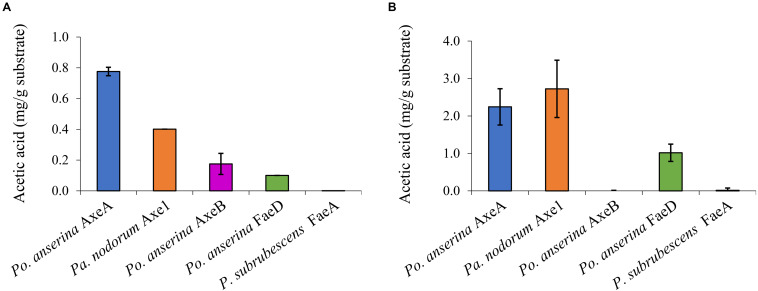
Acetic acid release from wheat arabinoxylan and corn oligosaccharides the selected CE1 enzymes or *P. subrubescens* FaeA. **(A)** Wheat arabinoxylan, **(B)** corn oligosaccharides. Each experiment was performed in triplicate. Standard deviations are shown as error bars.

Wheat arabinoxylan and COS were used as substrates to determine the ability of the enzymes to release acetic acid from plant biomass (Figure 4). WAX and COS contain about 1 and 3.2 mg acetic acid per one g polysaccharide, respectively. *Po. anserina* AxeA, *Pa. nodorum* Axe1 and *Po. anserina* FaeD released acetic acid from both WAX and COS, whereas *Po. anserina* AxeB only released acetic acid from WAX. The highest acetic acid release was detected for *Po. anserina* AxeA and *Pa. nodorum* Axe1 from WAX (78%) and COS (85%), respectively. A non-CE1 FAE from *Penicillium subrubescens* FaeA (Dilokpimol et al., 2016) was used for comparing the degrading ability of CE1 enzymes. *P. subrubescens* FaeA showed no release of acetic acid from either WAX or COS.

**FIGURE 5:**
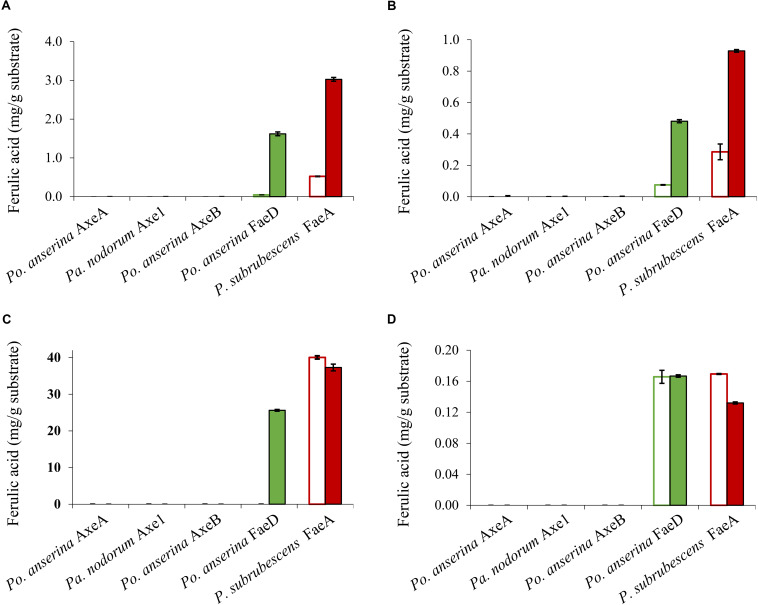
Ferulic acid release from plant biomass using the selected CE1 enzymes or *P. subrubescens* FaeA. **(A)** Wheat arabinoxylan, **(B)** wheat bran, **(C)** corn xylooligosaccharides, **(D)** sugar beet pectin. Empty bars indicate reactions with non-treated plant biomass, filled bars indicate reactions with pre-treated biomass (with xylanase for wheat arabinoxylan, wheat bran, corn xylooligosaccharides or arabinose and galactanase for sugar beet pectin) prior to incubation with CE1 enzymes or *P. subrubescens* FaeA. Each experiment was performed in triplicate. Standard deviations are shown as error bars.

Wheat arabinoxylan, WB, CX and SBP were used as substrates to verify the ability of the enzymes to release ferulic acid from plant biomass (Figure 5), as these substrates contain about 3, 1, 40, and 1.9 mg total ferulic acid per one g polysaccharide, respectively (Dilokpimol et al., 2017). *Po. anserina* FaeD released ferulic acid from all tested substrates, whereas none of the others showed any FAE activity. *Po. anserina* FaeD released the highest amount (64%) of ferulic acid from CX which was pre-treated with xylanase and no ferulic acid was detected when CX was not pre-treated with xylanase. *Po. anserina* FaeD also released a higher amount of ferulic acid from pre-treated WAX and WB with xylanase (54 and 48%, respectively) than those without xylanase pre-treatment (2 and 8%). *P. subrubescens* FaeA released a higher amount of ferulic acid from WAX, WB and CX than *Po. anserina* FaeD, and released all ferulic acid from pre-treated WAX and WB. The incubation of SBP with arabinanase and galactanase did not improve the release of ferulic acid by *Po. anserina* FaeD and *P. subrubescens* FaeA, and both enzymes only released less than 10% ferulic acid from SBP.

## Discussion

The CE1 is a diverse family and the fungal CE1 members can be classified into five subfamilies (CE1_SF1-SF5) (Figure 1 and Supplementary Figure 1). Multiple sequence alignment of members from CE1_SF1 and SF2 showed that these enzymes share an amino acid sequence similarity above 70%, which indicated that both CE1_SF1 and SF2 are more related to each other than to the other subfamilies. The same catalytic triad (Ser/Asp/His), the signature motif (G-X-S-X-G) and the shared common node suggested that these two subfamilies are likely to evolve from the same ancestor (Koseki et al., 1997; Komiya et al., 2017; Mäkelä et al., 2018). However, both subfamilies target substrates with a different molecular structure. So far, only a limited number of fungal CE1 enzymes have been characterized (Table 1), which hinders to explain the different activity and evolution relationship between CE1_SF1 and SF2.

In this study, we selected six ascomycete candidates from the uncharacterized branches in CE1_SF1 and SF2 to further explore the differences between CE1_SF1 and SF2. Four of these (*Po. anserina* AxeA, *Po. anserina* AxeB, *Pa. nodorum* Axe1 and *Po. anserina* FaeD) could be successfully produced in *P. pastoris* and exhibited different properties. Both *Pa. nodorum* and *Po. anserina* belongs to the Phylum Ascomycota, Subphylum Pezizomycotina, while the first is classified in the Class Dothideomycetes, the latter is part of the Class Sordariomycetes. Based on the AXE activity assay, *Po. anserina* AxeA, *Po. anserina* AxeB and *Pa. nodorum* Axe1 from CE1_SF1 showed the highest activity toward MUB-acetate, while *Po. anserina* FaeD from CE1_SF2 showed around 10-fold less activity on this substrate (Table 2). Even though all four enzymes showed activity toward AXE substrates, only *Po. anserina* FaeD also de-esterified the FAE substrates with broad substrate preference. Most characterized FAEs from this subfamily showed broad substrate specificity including FaeB, AsFae, MtFae1a, and ClFaeB2, supporting that this is one of the unique features of the FAEs in CE1 (Kroon et al., 2000; Kühnel et al., 2012; Topakas et al., 2012; Dilokpimol et al., 2016, 2018).

All enzymes could release acetic acid from both WAX and COS, except for *Po. anserina* AxeB for which the release of acetic acid was only detected from WAX (Figure 4). This could result from the different position of the acetylation in the substrates. Acetylation in commelinid monocots including wheat generally occurs at the *O*-2 and/or *O*-3 position of the xylose residues of the xylan backbone (Puls, 1997; Appeldoorn et al., 2010). It has been shown earlier that AXEs from CE1 regioselectively cleave the substituents in the *O*-2 and *O*-3 position, and deacetylate the *O*-2 position faster than the *O*-3 position (Altaner et al., 2003). However, COS contains more than half of the acetyl group attached to the *O*-2 position of the xylose backbone, while the same xylose is also substituted with a monomeric α-L-arabinosyl residue at the *O*-3 position (Appeldoorn et al., 2013). Because *Po. anserina* AxeA and *Pa. nodorum* Axe1 released over 75% of total acetic acid, our results also indicated the potential ability of these enzymes to attack the dense acetyl substitution on the xylan backbone in COS.

In contrast, only *Po. anserina* FaeD could release ferulic acid from the feruloylated substrates (Figure 5). As mentioned before, feruloylation in commelinid monocots mainly occurs at the *O*-5 position on arabinosyl residues of xylan (Smith and Hartley, 1983; Saulnier et al., 1995; Wende and Fry, 1997; Schendel et al., 2016), while in pectin it mainly occurs at the *O*-2 and/or *O*-5 positions of arabinan side chains and at the *O*-6 position of D-galactosyl residues in (arabino-)galactan of rhamnogalacturonan I (Carpita, 1996; Levigne et al., 2004; Harris, 2005; Harris and Trethewey, 2010). *Po. anserina* FaeD was shown to release more ferulic acid from commercial xylanase-treated xylan substrates (WAX, WB, and CX) than non-treated ones, from which it released only small amounts of ferulic acid. In contrast, pre-treatment of SBP with arabinanase and galactanase did not improve the release of ferulic acid by *Po. anserina* FaeD and less than 10% of ferulic acid was released from intact and pre-treated pectin. Analysis of ferulic acid release from xylan and SBP substrates indicated that pre-treatment had a larger effect on *Po. anserina* FaeD activity on xylan substrates than SBP. This difference indicated that *Po. anserina* FaeD is mainly active on *O*-5 feruloylated xylooligosaccharides and much less on other substitutions. *Po. anserina* FaeD from CE1_SF2 showed a high preference for xylan substrates, which resembles FAEB from *Penicillium funiculosum* (Kroon et al., 2000) and FaeC from *Aspergillus niger* (Dilokpimol et al., 2017). *P. subrubescens* FaeA, a non-CE1 FAE control, did not release acetic acid from WAX or COS, but could efficiently release ferulic acid from WAX, WB, and CX. Earlier, an FAE from *Aspergillus oryzae* (AoFae) was shown to possess dual activity (Tenkanen et al., 1991). AoFae also belongs to CE1 based on sequence homology of the N-terminal amino acid sequence (Tenkanen et al., 2003). However, based on the hydrolysis pattern toward different monoacetylated and monoferuloylated *p*-nitrophenyl glycosides, AoFae was later suggested to be a non-specific acetyl esterase (Puchart et al., 2006). In the same study, the authors also showed that some FAEs could also release acetyl residue from the same substrates with specific positional specificity, but with at least two orders of magnitude lower (Puchart et al., 2006). This aspect should be validated further for these new enzymes to monitor the de-esterified positions.

Based on the multiple sequence alignment (Supplementary Figure 3) between CE1_SF1 and SF2, Trp160 was highly conserved among CE1_SF1 members, whereas the corresponding amino acids in CE1_SF2 can be an Ala, Ser, Pro, Gln or Thr (Supplementary Table 1). Recently, a crystal structure of an *Aspergillus luchuensis* (formerly *A. awamori*) *Al*AXEA (PDB code 5X6S) showed Trp160 controls the substrate specificity of AXE in CE1 (Komiya et al., 2017). When replacing Trp160 to Ala, Ser, or Pro, the mutants showed significant FAE activity. Trp160 in *Al*AXEA is corresponding to an Ala in *Po. anserina* FaeD supporting that the expanded substrate specificity of an AXE to FAE and the dual activity is potentially influenced by this amino acid.

To investigate the pH and temperature profile of these recombinant enzymes, we used MUB-acetate as a substrate for AXE activity. All candidates showed optimum pH in a neutral range (pH 6.0 to 7.0) and an optimum temperature between 30 and 50°C, which are similar to most other characterized CE1_AXE enzymes (Koseki et al., 1997, 2006; Chung et al., 2002; Pouvreau et al., 2011; Huy et al., 2013;Table 1). Surprisingly, they all showed excellent pH stability over a broad pH range, retaining more than 80% residual activity after an incubation at pH 6.0–9.0 for 2 h, which showed a similar pH stability range to *Aspergillus luchuensis Al*AXEA (Koseki et al., 1997; Komiya et al., 2017). It should be noted that at pH more than 7.0, the ester-linkages are alkali labile and tend to degrade easily. Hence, to determine the pH stability of AXEs, we used culture filtrate from *P. pastoris* harboring pPicZαA plasmid without insertion as negative control, from which the residual activity was deduced. Using methyl ferulate as a substrate for FAE activity, *Po. anserina* FaeD also showed optimum pH at 7.0 and optimum temperature at 50°C, these properties are within the range of other reported fungal FAEs, mainly with activity at pH 4–8 and temperature 30–65°C (Table 1; Crepin et al., 2003; Topakas et al., 2007, 2012; Kühnel et al., 2012). It also showed a high pH stability over a broad pH range from pH 3.0 to 10.0, which is more superior than most reported CE1 enzymes (Table 1). The CAZymes from *Pa. nodorum* (synonyms: *Stagonospora*/*Septoria*/*Phaeosphaeria* nodorum) have not been characterized in much detail, mainly because it is a major pathogen of wheat and related cereals (Downie et al., 2018). Several CAZymes from *Po. anserina* were previously reported, e.g., *Pa*Man5A and *Pa*Man26A mannanases, *Pa*Xyn11A xylanase, and *Pa*Abf51A and *Pa*Abf62A arabinofuranosidases were active in a range of pH 3–7 and 5–75°C (Couturier et al., 2011), while *Pa*Cel6A-C were active in a range of pH 4–9 and 25–65°C (Poidevin et al., 2013). The biochemical properties of different enzymes from the same strain can be vastly different also depending on the function of the enzyme, which most of the enzymes in this study are quite stable at high pH. The alkaline tolerance of the newly characterized enzymes is of interested for many bio-industrial applications particularly for the alkali pre-treated plant biomass.

## Conclusion

CE1_SF1 and SF2 are related, even though the characterized enzymes from the first possess AXE activity and the ones from latter possess FAE activity. So far no additional activity was detected in these subfamilies except for the dual activity. A novel dual FAE/AXE activity enzyme was identified from CE1_SF2, which showed promising industrial applications because of its broad substrate specificity. To further understand the functional features and physiological role of individual enzymes, the positional specificity of these new enzymes should be further investigated. The phylogenetic analysis and multiple sequence alignment supported the evolutionary relationship between CE1_SF1 and SF2, and identified possible amino acids that control the AXE or FAE activity between these two subfamilies. Moreover, these novel fungal AXE and FAE enzymes showed a great alkaline tolerance and can selectively release acetic acid and ferulic acid from different plant-based biomass making them attractive for various biotechnological applications.

## Data Availability Statement

All datasets generated for this study are included in the article/Supplementary Material.

## Author Contributions

XL, KG, and AD conducted the main experiments, analyzed the data, and drafted the manuscript. MF, EU, and MK contributed to HPLC analysis for acetic acid. XL, KG, and SL contributed to phylogenetic analysis. AD and RV contributed to data interpretation and commented on the manuscript. RV conceived and supervised the overall project. All authors commented on the manuscript.

## Conflict of Interest

The authors declare that the research was conducted in the absence of any commercial or financial relationships that could be construed as a potential conflict of interest.

## Footnotes

1 https://genome.jgi.doe.gov/mycocosm/home

2 http://www.cazy.org/

3 http://www.cbs.dtu.dk/services/SignalP/

4 https://mafft.cbrc.jp/alignment/server/

5 http://espript.ibcp.fr/ESPript/ESPript/

6 https://www.megasoftware.net/

7 http://www.cbs.dtu.dk/services/NetNGlyc/

8 https://www.bioinformatics.org/sms2/protein_mw.html
