# Supplementary material for: Functional Validation of Two Fungal Subfamilies in Carbohydrate Esterase Family 1 by Biochemical Characterization of Esterases From Uncharacterized Branches
Source: Front Bioeng Biotechnol. 2020 Jun 26;8:694. doi: 10.3389/fbioe.2020.00694 (PMC7332973; doi:10.3389/fbioe.2020.00694)
Supplement: Supplementary file 1 [file Image_1.pdf]

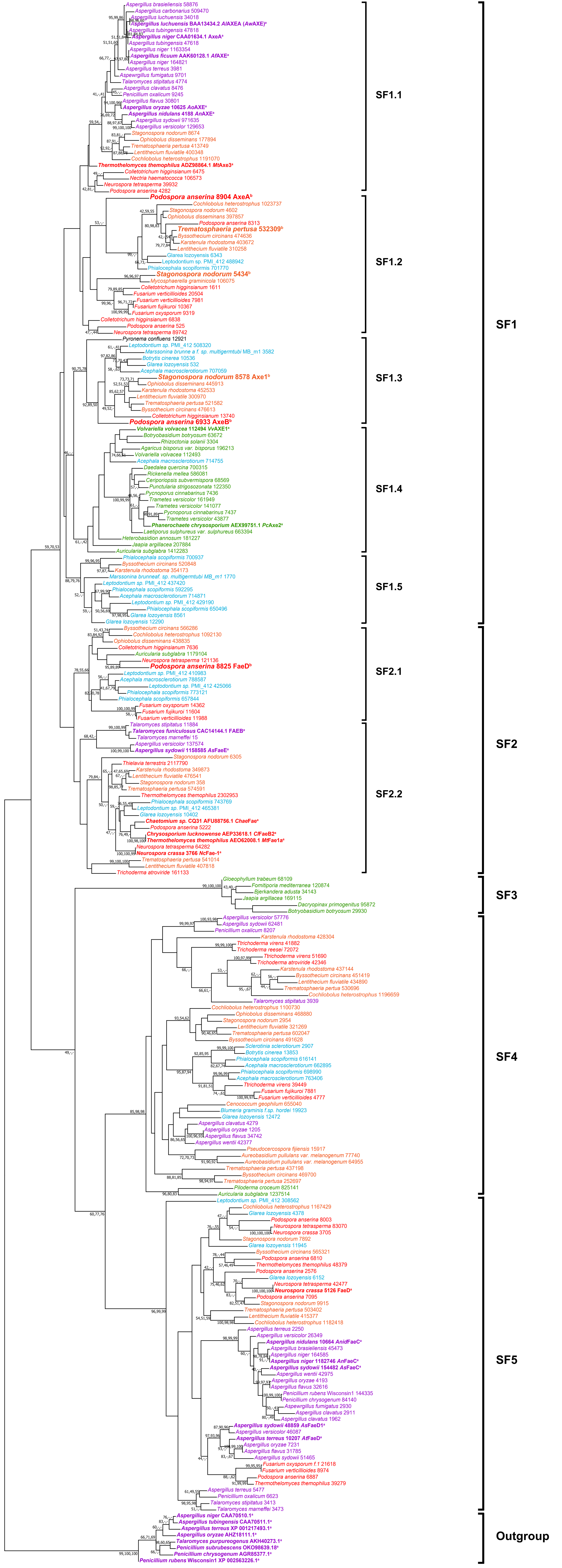

Supplementary Figure 1. The full phylogenetic tree using total 247 fungal CE1 sequences. The phylogenetic analysis was performed by maximum likelihood (ML) implemented in MEGA7 (Kumar et al., 2016) with 95% partial deletion of gaps and the Poisson correction distance of substitution rates. The main branches/subfamilies were collapsed. Statistical support for phylogenetic grouping was estimated by 500 bootstrap re-samplings, only the bootstrap above 40% were shown on the branches, also of a neighbor-joining (NJ) and minimal evolution (ME) tree using the same dataset (order: ML, NJ, ME). Eight FAEs from subfamily 7 (Dilokpimol et al., 2016) were used as an outgroup. SF, subfamily. The purple highlighted eurotiomycetes, the orange highlighted dothidiomycetes, the red highlighted sordariomycetes, the blue highlighted leotiomycetes, the green highlighted agaricomycotina and the black one is pezizomycetes.
