# Supplementary material for: Functional Validation of Two Fungal Subfamilies in Carbohydrate Esterase Family 1 by Biochemical Characterization of Esterases From Uncharacterized Branches
Source: Front Bioeng Biotechnol. 2020 Jun 26;8:694. doi: 10.3389/fbioe.2020.00694 (PMC7332973; doi:10.3389/fbioe.2020.00694)
Supplement: Supplementary file 2 [file Image_2.pdf]

<table>
<tr><td>Fungi</td><td colspan="2">Po. anserina</td><td colspan="2">T. pertusa</td><td colspan="2">Pa. nodorum</td><td rowspan="3">Marker</td><td colspan="2">Pa. nodorum</td><td colspan="2">Po. anserina</td><td colspan="2">Po. anserina</td></tr>
<tr><td>Acc. No.</td><td colspan="2">Podan2|
8904</td><td colspan="2">Trepe1|
532309</td><td colspan="2">Stano2|
5434</td><td colspan="2">Stano2|
8578</td><td colspan="2">Podan2|
6933</td><td colspan="2">Podan2|
8825</td></tr>
<tr><td>Enzyme</td><td colspan="2">AxeA</td><td colspan="2">N.P.</td><td colspan="2">N.P.</td><td colspan="2">AxeA</td><td colspan="2">AxeB</td><td colspan="2">FaeD</td></tr>
<tr><td>Endo H</td><td>–</td><td>+</td><td>–</td><td>+</td><td>–</td><td>+</td><td>(kDa)</td><td>–</td><td>+</td><td>–</td><td>+</td><td>–</td><td>+</td></tr>
</table>

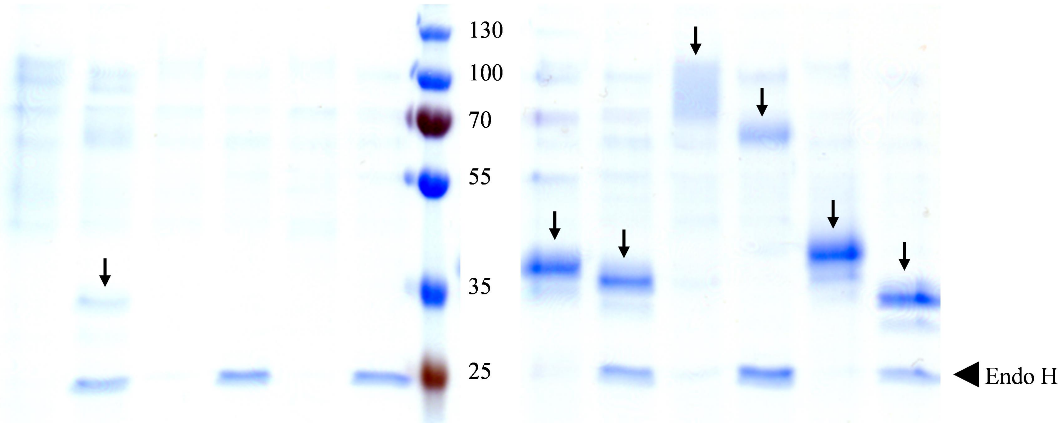

Supplementary Figure 2. Recombinant protein production of the selected candidates with (+) and without (-) endoglycosidase H (Endo H) treatment. Black arrows indicate the target bands of candidates and black triangle indicates the endoglycosidase H band. Acc. No., Accession number of the selected candidates. N.P., no production.
