# Supplementary material for: Functional Validation of Two Fungal Subfamilies in Carbohydrate Esterase Family 1 by Biochemical Characterization of Esterases From Uncharacterized Branches
Source: Front Bioeng Biotechnol. 2020 Jun 26;8:694. doi: 10.3389/fbioe.2020.00694 (PMC7332973; doi:10.3389/fbioe.2020.00694)
Supplement: Supplementary file 3 [file Image_3.pdf]

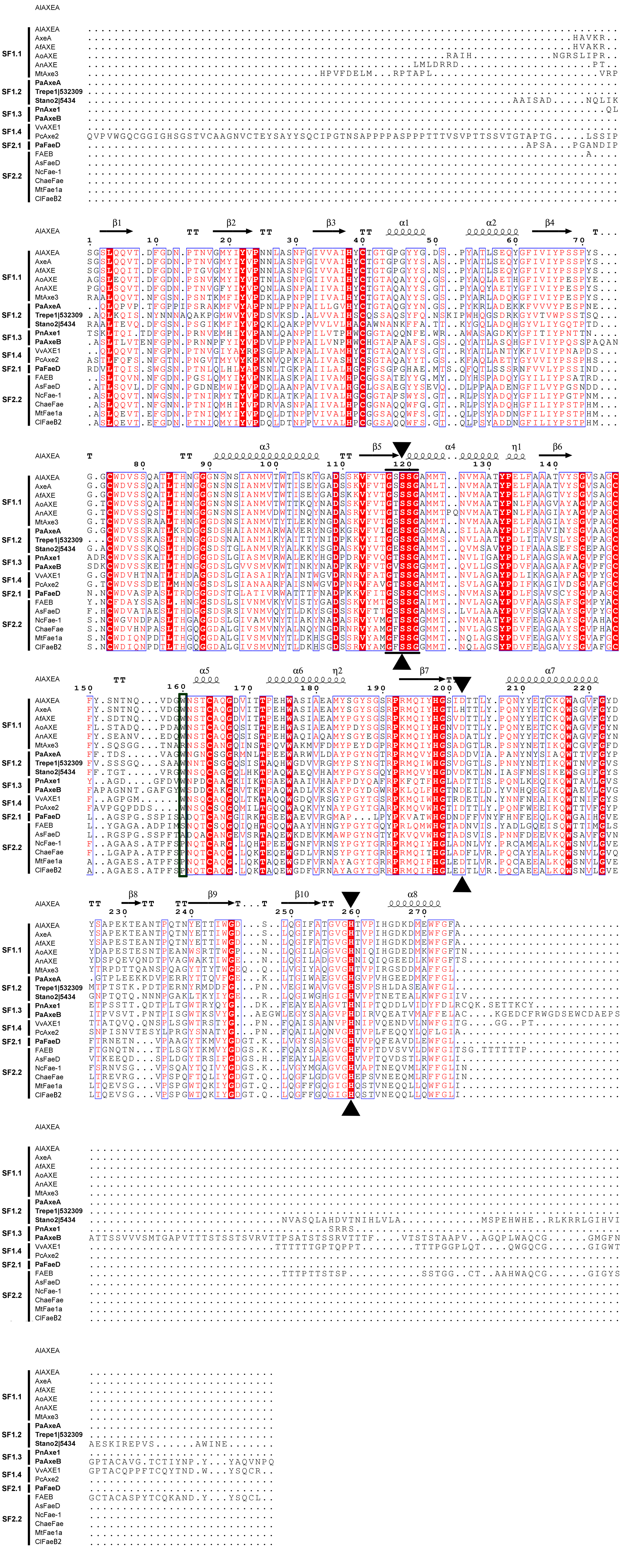

Supplementary Figure 3. Amino acid sequences alignment among the characterized fungal CE1_SF1 and SF2 and the selected candidates. *Aspergillus ficuum* AfAXE (Chung et al., 2002), *Aspergillus luchuensis* AlAXEA (Koseki et al., 1997; Komiya et al., 2017), *Aspergillus niger* AxeA (De Graaff et al., 1992), *Aspergillus nidulans* AnAXE (Bauer et al., 2006), *Aspergillus oryzae* AoAXE (Koseki et al., 2006), *Aspergillus sydowii* AsFaeE (Dilokpimol et al., 2018), *Chaetomium sp.* CQ31 ChaeFae (Yang et al., 2013), *Neurospora crassa* NcFae-1 (Crepin et al., 2003), *Parastagonospora nodorum* PnAxe1, *Penicillium funiculosum* FAEB (Kroon et al., 2000), *Phanerochaete chrysosporium* PcAxe2 (Huy et al., 2013), *Podospora anserina* PaAxeA, *Podospora anserina* PaAxeB, *Podospora anserina* PaFaeD, *Stagonospora nodorum* 5434, *Thermothelomyces thermophiles* MtFae1a (Topakas et al., 2012), *Thermothelomyces thermophiles* ClFaeB2 (Kühnel et al., 2012), *Thermothelomyces thermophiles* MtAxe3 (Pouvreau et al., 2011), *Trematosphaeria pertusa* 532309, *Volvariella volvacea* Vvaxe1 (Ding et al., 2007). Black triangles indicate (putative) catalytic triad (Ser/Asp/His). The line indicates the comment G-X-S-X-G motif. Green box indicates Trp160 in AlAXEA. Names in bold indicate the selected candidates for this study.
